# A study on the mitigating effect of different music types on motion sickness based on EEG analysis

**DOI:** 10.3389/fnhum.2025.1636109

**Published:** 2025-09-03

**Authors:** Yilun Li, Yue Li, Yan Li, Bingjie Luo, Bangbei Tang, Qizong Yue

**Affiliations:** ^1^School of Music and Dance, Henan Institute of Science and Technology, Xinxiang, China; ^2^School of Intelligent Manufacturing Engineering, Chongqing University of Arts and Sciences, Chongqing, China; ^3^Department of Physiology, Army Medical University, Chongqing, China; ^4^China Music Mental Health Institute, Southwest University, Chongqing, China

**Keywords:** electroencephalographic data, motion sickness recognition, relief methods, music therapy, machine learning

## Abstract

**Introduction:**

Motion sickness often causes passengers to experience negative emotions such as tension, which in turn triggers symptoms like dizziness and nausea, seriously affecting the travel experience of passengers. Previous studies have shown that music can alleviate negative emotions such as tension, but its effect on motion sickness remains unclear, and the differences in the alleviation effect of different types of music on motion sickness need to be quantitatively evaluated.

**Methods:**

We collected Electroencephalogram (EEG) data from 30 subjects in a simulated driving environment and constructed a motion sickness recognition model by combining time-and frequency-domain features (mean, variance, skewness, kurtosis, power spectral density) with classification algorithms. The model achieved accurate identification of passenger motion sickness states. Based on this model, the intervention effects of four types of music (joyful, sad, stirring, and soft) on motion sickness were further evaluated and compared with the control group (taking natural recovery measures).

**Results:**

The results showed that soft and joyful music had better intervention effects (average reduction of 56.7 and 57.3%, respectively), followed by passionate and sad music (average reduction of 48.3 and 40%, respectively), among which the alleviation effect of sad music was lower than that of the control group (average reduction of 43.3%). In addition, it was verified that the EEG Kolmogorov-Chaitin complexity in the occipital region was significantly negatively correlated with the motion sickness grade *p* = −0.625, *p* < 0.005).

**Discussion:**

The study suggests that personalized music intervention strategies may effectively alleviate motion sickness symptoms of passengers, thereby increasing cabin comfort and improving the travel experience of passengers.

## 1 Introduction

With the advancement of autonomous driving technology to SAE Level 3, human-machine shared control has emerged as the predominant operational paradigm (He et al., 2024). In this configuration, drivers collaborate dynamically with the autonomous system, relinquishing continuous vehicular control. While this transition enhances driving convenience and safety, it simultaneously introduces novel kinesthetic challenges for occupants, particularly a marked elevation in motion sickness incidence. Empirical evidence indicates that passengers experience significantly higher susceptibility to motion sickness compared to drivers (Smyth et al., 2018). During mixed-mode operation (alternating between manual and assisted driving), occupants frequently develop motion sickness when kinematic vehicle parameters (e.g., longitudinal/lateral acceleration, yaw rate) conflict with visual cues, resulting in vestibulo-ocular mismatch.

Currently, the measurement methods of motion sickness are mainly divided into subjective measurement and objective measurement (Chang et al., 2020). Subjective measurements are based on relevant questionnaires, which are filled out verbally or independently to obtain the patient’s motion sickness rating, while objective measurements rely on physiological signal acquisition equipment to collect objective physiological and behavioral data from patients and correlate them with the user’s subjective motion sickness ratings, thus exploring the relationship between objective parameters and motion sickness. Subjective measures of motion sickness are commonly used, including the Pensacola Motion Sickness Questionnaire (MSQ; Graybiel et al., 1965), the Simulator Sickness Questionnaire (SSQ; Kennedy et al., 1993), and the Misery Scale (MISC; Bos et al., 2005).

A large number of studies have been devoted to the mitigating modulation of motion sickness, mainly focusing on pharmacological management and sensory interventions, and it has been shown that there is expression of cholinergic M1, M2, and M5 receptor subtypes in the vestibular organ and vestibular ganglion (Li et al., 2007), with the M1 and M5 receptors functioning as postsynaptic excitatory receptors. Based on this finding, the anti-motion sickness effect of scopolamine may originate from its specific blocking effect on M1 and M5 receptor subtypes (Zhang et al., 2016). Hongri et al. (2025) developed a motion sickness model using rotational stimulation and assessed the efficacy of Tianmu ultrafine powder in alleviating symptoms through behavioral indices (e.g., motion sickness response index, balance beam test, spontaneous activity test). Their results demonstrated that this powder significantly reduced motion sickness in mice without adverse effects (Hongri et al., 2025). Xiang (2024) investigated the efficacy of intradermal needle therapy in alleviating motion sickness. Rotational motion was used to induce motion sickness in the subjects, with blood pressure and pulse rate as indicators. The results showed that intradermal needles stimulated at a frequency of 60 times/min were more effective in improving the symptoms and signs of rotation-induced motion sickness, suggesting that higher stimulation frequencies may be closer to the optimal treatment dose and can achieve more satisfactory treatment outcomes (Xiang, 2024). While existing research has predominantly focused on optimizing motion sickness recognition algorithms and developing olfactory-or tactile-based interventions, the relationship between auditory stimuli (particularly music genres) and motion sickness in driving environments remains underexplored.

To address these issues, this study focuses on investigating the effects of different types of music on motion sickness. By constructing a motion sickness identification model based on electroencephalographic signals, we systematically evaluate the differential regulatory effects of four types of music (joyful music, sad music, stirring music, and soft music) on motion sickness.

The main contributions of this work can be summarized as follows:

(1) The system quantified the differentiated intervention effects of four types of music on motion sickness and found that joyful music and soft music had better alleviating effects on motion sickness (57.3 and 56.7%, respectively), providing empirical evidence for cabin music intervention for motion sickness.
(2) The recognition model based on electroencephalographic signals (particularly the occipital lobe BPNN model, with an accuracy rate of 85.6%) provides an objective measurement tool for evaluating the effectiveness of music intervention.
(3) Extraction and analysis of the complexity of KC in the occipital lobe confirmed a certain correlation with motion sickness, proving that it can be used as an assessment indicator for motion sickness.

## 2 Materials and methods

### 2.1 Experimental scenario

Since this experiment requires the collection of EEG data from subjects in the motion sickness state, and the induction of motion sickness symptoms in the actual road environment may lead to a decrease in driving maneuvering ability, which is a safety hazard. Therefore, in this study, a driving simulation experiment was used instead of a real-road experiment to induce motion sickness symptoms and collect EEG data from the subjects. The main advantages of the simulator are that it is safe and avoids the risk of driving due to motion sickness, and the experimental environment can be controlled to precisely adjust the parameters of the visuomotor stimuli to induce different degrees of motion sickness. It has been shown that the visual-vestibular conflict effect generated by driving simulators is physiologically similar to the real motion environment and can effectively induce typical motion sickness symptoms (Bronstein et al., 2020). Based on this, the present study was conducted to collect EEG data under motion sickness through a driving simulator experimental platform.

In this study, Forza Horizon 5 software developed on the EA platform and Lestar V99 driving simulator are used to build a driving simulation environment, which can simulate the road traffic environment of the driver in the real driving process (Ali et al., 2020), and contains the vehicle operating system, the image display system, and the sound system in three parts, and the simulator has certain assisted driving functions, which can be good simulation of the current assisted driving and manual driving synthetic environment. Driving and manual driving synthetic environment. The simulator’s display screen has a width-to-height ratio of 16:9, with a horizontal field of view (FOV) of 83° and a vertical FOV of 53°. The vehicle control system is equipped with a steering wheel, gearshift, accelerator pedal, brake pedal, and clutch. The visual display system includes an LCD screen that provides a first-person driving perspective, while the audio system delivers surround sound effects during the simulated driving process. The EEG data acquisition device operates at a sampling rate of 500 Hz. The 64-channel EEG electrodes are positioned according to the international 10–10 system, with CPz and End electrodes serving as reference and ground, respectively. Throughout the experiment, electrode impedance is maintained below 5 kΩ. The entire experimental process is programmed using E-Prime 3, which can be connected to the EEG equipment to synchronize marking. Music control is also achieved through E-Prime 3 by connecting to headphones for playback.

**Figure 1:**
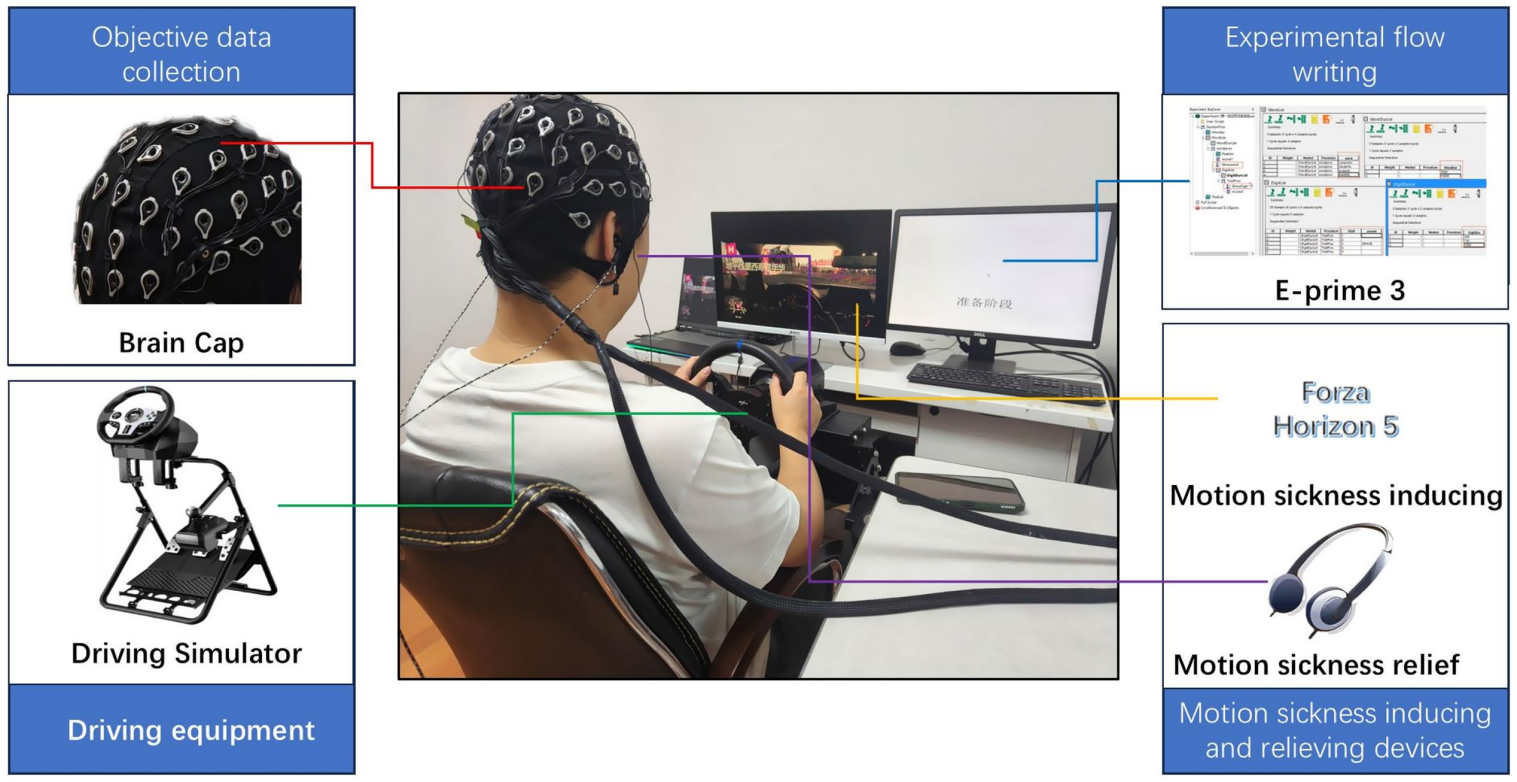
Experimental scenario.

The experimental setup scene is shown in Figure 1. The environment in the laboratory was always well ventilated and well lit.

### 2.2 Initial screening of simulator roads

Forza Horizon 5 software has built-in rich and real road sections to choose from. In order to achieve better motion sickness inducing effects on the subjects during the formal experiments, the built-in roads are first screened, taking into account the experimental needs, the susceptibility of the subjects to motion sickness, and the degree of driving proficiency, and are screened in accordance with the following principles:

(1) The road trip should be appropriate, the road trip is too short to meet the set experimental time requirements, the trip is too long will lead to fatigue and interfere with the motion sickness induced experiment.
(2) The road should have good motion sickness inducing effect, which can ensure the successful induction of most subjects.
(3) The complexity of the road should be appropriate; if the road complexity is too low, the motion sickness inducing effect will be poor, which will affect the accuracy of the experiment, and at the same time, the road conditions are too single, which will easily cause fatigue; while if the complexity is too high, the subjects with low driving skill may frequently have collisions and drive out of the road area, which will interfere with the experiment.

In accordance with the above principles, 10 roads with different road complexity, road length, and different surrounding landforms (rainforest, desert, mountain, coast, etc.) were selected in the software, and all of them required more than 5 min of traveling time to complete. These 10 roads will be used for subsequent experimentalists to analyze and screen the effectiveness of road sickness induction.

In order to assess the 10 roads initially screened in the driving simulator as described above, the MISC, Karolinska Sleepiness Scale (KSS; Kaida et al., 2006), and the 7-level Likert Scale (Capuano et al., 2016) were used to record the subjects’ motion sickness level, fatigue level, and road complexity during the driving task, respectively. The MISC, KSS, and Likert Scale used are shown in Tables 1–3.

**Table 1:** MISC scale.

Symptomatic	Motion sickness	Level
No symptoms.		0
I’m a little uncomfortable, but I do not have any obvious symptoms.		1
Dizziness, feeling cold/hot, headache, upset stomach, upset throat, increased sweating, blurred vision, Yawning, hiccups, tiredness (fatigue), increased saliva production, but not nausea	Seemingly tangible or intangible	2
	Mildly	3
	Moderately	4
	Severe	5
Nausea	Mildly	6
	Moderately	7
	Severe	8
	Borderline dry heaving	9
Vomiting		10

**Table 2:** Karolinska sleepiness scale.

Degree of sleepiness	Score
Fully conscious state	1
Very lucid state	2
Sober	3
More awake	4
Between wakefulness and sleepiness	5
More sleepy	6
Drowsy but alert	7
Drowsy but less able to maintain alertness	8
Extremely sleepy and wanting to go to sleep	9

**Table 3:** 7-level Likert scale.

Descriptive	Define	Score
Almost no maneuvering, no obstacles on the road	Maximum simplicity	1
Very little attention is required and the operation is fully automated.	Very simple	2
Occasional attention to road conditions is required, but handling is stress-free.	Simpler	3
Requires steady attention and a clear need to operate.	Moderate	4
High-frequency operation requiring a high degree of concentration.	Sticky	5
Complex road conditions with low operational tolerance.	Very difficult	6
It’s almost impossible to complete the driving task.	Great difficulty	7

Forty volunteers were recruited to participate in the simulator road screening experiment, including 22 males and 18 females, and the specific information of the volunteers is shown in Table 4.

**Table 4:** Volunteer age, driving age details.

Age, years driving	Average value	Standard deviation	Upper quartile
Age (years)	27.8	6.1	26
Driving experience (years)	5.0	3.0	4.5

**Figure 2:**
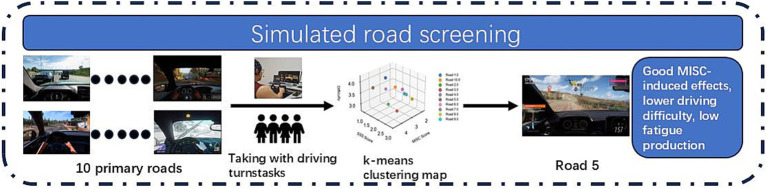
Simulator road screening experimental procedure.

During the experiment, the subjects completed the driving tasks of 10 roads sequentially according to the requirements, and every time they completed a driving task, they filled in the MISQ scale and Caroline Sleepiness Scale for that road, and when the level of motion sickness reached 3 or more, it was regarded as a successful induction; in order to avoid too much intervention of the cumulative effect of motion sickness, after the completion of filling in the scales, the subjects would be free to move around for 3 min in order to alleviate the effect of motion sickness and wake up the brain, and after 3 min After 3 min, the subjects will continue to complete the next road driving task. The screening process is shown in Figure 2.

**Figure 3:**
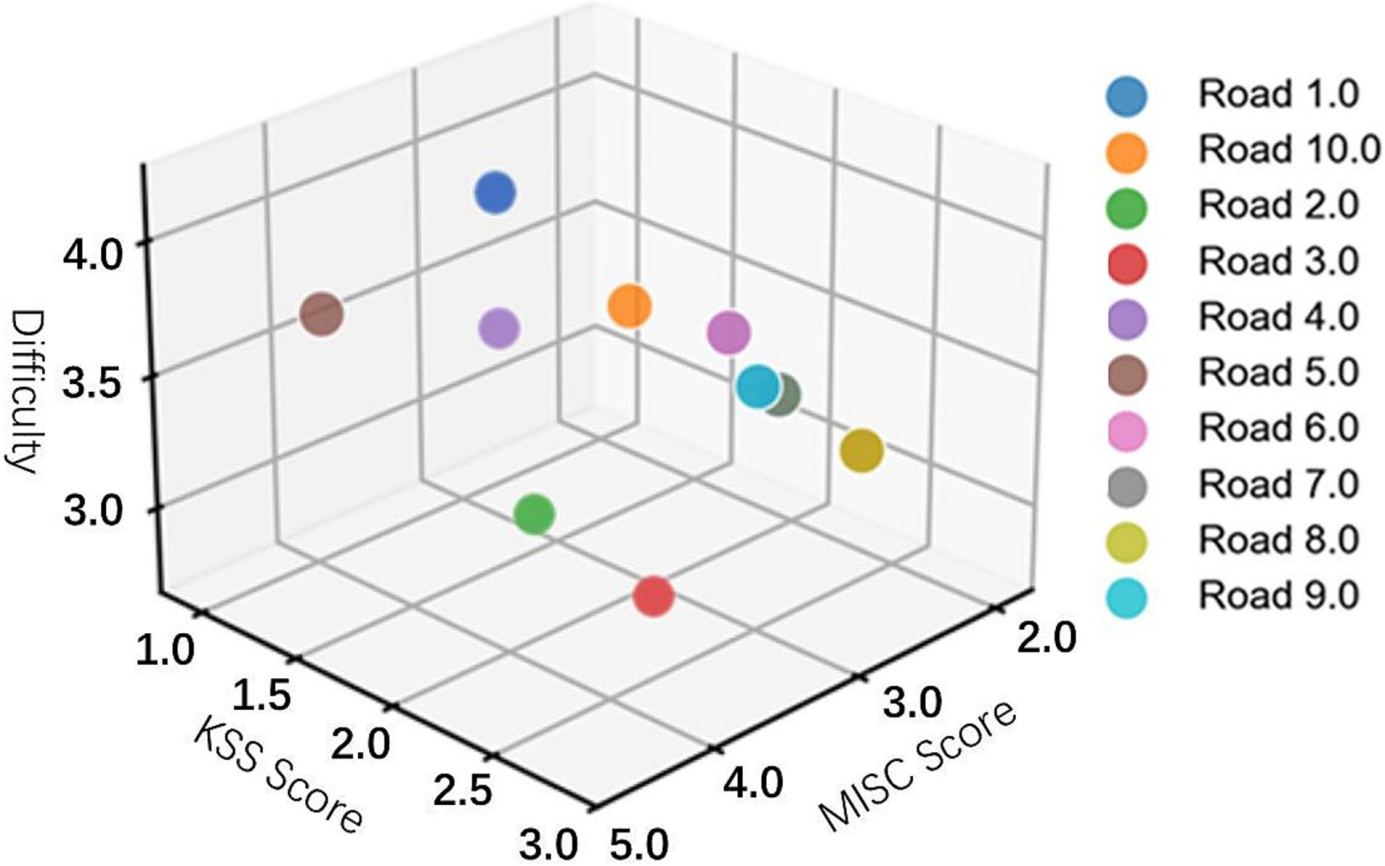
Simulated roadway k-means clustering map.

The 10 roads were numbered and the data from the experiment were collected to present the performance of the 10 roads in the three dimensions using k-means clustering diagram. As shown in Figure 3.

According to the aforementioned screening principles, it can be seen that road 2, road 3 and road 5 have better motion sickness inducing effect, but road 2 and road 3 are easy to make people tired, so the comprehensive consideration, road 5 was selected as the subsequent experimental motion sickness inducing material.

### 2.3 Experimental procedure

#### 2.3.1 Subjects were screened for susceptibility to motion sickness

Due to individual differences in sensitivity to motion stimuli and to visual conflict-inducing schemes such as driving simulators, in order to ensure that the experiment can effectively induce observable motion sickness symptoms and control the variability brought about by individual differences, and to ensure the efficient conduct of the experiment, subjects with moderate susceptibility to motion sickness were selected for the follow-up music-relieving experiments in this study.

Translated with DeepL.com (free version) all questionnaire screeners are required to meet the following criteria:

(1) Corrected visual acuity ≥ 1.0 (Snellen 20/20), no color blindness or color deficiency (by Ishihara test), and no history of recent ophthalmic surgery.
(2) No history of mental illness, cardiovascular disease, vestibular dysfunction or syncope, and no medication affecting vestibular function 48 h before the experiment.
(3) Pure tone audiometry ≤ 25 dB HL, no tinnitus or balance disorders.

Motion sickness susceptibility is generally assessed using the Motion Sickness Susceptibility Questionnaire (MSSQ; Lukacova et al., 2023), which is an effective predictor of susceptibility to motion sickness in a laboratory setting by assessing the frequency and severity of motion sickness in individuals who have ridden various types of transportation in the past. However, some of the questions in the traditional questionnaire (e.g., amusement park rides: roller coasters, etc.) have limited applicability in the Chinese population, so the MSSQ modified by Leilei Pan of the Naval Medical University was used as the instrument in this study. Therefore, this study used the MSSQ modified by Pan Leilei of Naval Military Medical University, which replaces amusement park rides with more suitable means of transportation for Chinese subjects (e.g., “bus bumps”) to increase the accuracy of the screening process (Pan Leilei and Ruirui, 2016).

The modified version of the MSSQ scale consists of a childhood questionnaire and an adulthood questionnaire, with the childhood questionnaire recording up to the age of 12 years and the adulthood questionnaire recording within the last 10 years, and the questionnaires are the same during both childhood and adulthood, i.e., Tables 5, 6. The susceptibility index calculation and grading criteria are then based on a modified version of the MSSQ-R3 formula:


(1) $$\text{MSSQ}-R3-A(B)=\frac{(2{\mathrm{TSS}}_{N}+{\mathrm{TSS}}_{V})\times 7}{\mathrm{NST}}$$

**Table 5:** Sample questionnaire on the number of rides on transportation or amusement rides.

Entertainment activities	Never (1 point)	1–4 times (2 points)	5–10 times (3 points)	**11 or more (4 points)**
Enclosed carriage				
Bus				
Trains				
Helicopter				
Boat				
Ship or ferry				
Trapeze				

**Table 6:** Nausea and vomiting on transportation or amusement rides and vomiting symptoms on transportation or amusement rides.

Entertainment activities	Never (1 point)	Basically not. (2 points)	Occasionally (3 points)	Non-Recurrent (4 points)	Always (5 points)
Enclosed carriage					
Bus					
Trains					
Helicopter					
Boat					
Ship or ferry					
Trapeze					

In the formula, MSSQ−R3−A(B) is the susceptibility index during childhood or adulthood, respectively, and the sum of the two is the total susceptibility index; ${\mathrm{TSS}}_{N}$ is the total nausea symptom score, ${\mathrm{TSS}}_{V}$ is the total vomiting symptom score, and NST is the number of types of rides on transportation or amusement rides.

The percentile was used to determine the level of susceptibility grades: mild susceptibility (<50%), moderate susceptibility (50–75%), and severe susceptibility (>75%), and a total of 112 subjects were screened, from which 30 subjects with moderate susceptibility to dizziness were screened to participate in the subsequent experiment. And before the beginning of the experiment, the data of individual characteristics of the subjects were recorded: height, age, height, and body mass as shown in Table 7.

**Table 7:** Information sheet for subjects.

Scene	Males	Females	Age	Driving experience
Twisty mountain road	16	14	27.9	4.6

#### 2.3.2 Motion sickness triggering and music modulation

Thirty subjects will be divided into six groups of five each, four of which will be moderated with four types of music after the induction of motion sickness (moderated group), one group will mark the EEG data after the motion sickness score reaches 2 (almost no motion sickness) that is the end of the group (baseline group) without moderation, and the last group will serve as a control group, which will be taken to meditate for 1 min after the normal completion of the simulated driving task induced by the motion sickness, without other interventions.

In order to better study visually induced dystonia, all subjects were told to refrain from consuming alcohol, caffeine, and nicotine for 48 h before the start of the experiment, and to get enough sleep. Prior to the start of the experiment, all subjects were required to fill out an informed consent form to ensure that they fully understood the objectives and specific tasks of the experiment.

During the experiment, subjects were asked to verbally report the level of motion sickness according to a simplified version of the Motion sickness scale (MSS), which is shown in Table 8. The reason why the traditional MISC was not used is that it has more gradations and is not suitable for subjects to quickly judge their own state during the experiment. The use of the MSS improves the efficiency of the experiment, reduces subject talking, and decreases interference with the EEG data.

**Table 8:** Motion sickness scale.

None	Almost no motion sickness	Mildly	Moderately	Serious
1	2	3	4	5

The specific experimental procedure, which can be divided into the following three stages:

Preparatory stage:

In order for the subjects to understand the symptoms associated with motion sickness and to ensure that they were in good physical condition before the driving phase, the subjects were first asked to fill in the SSQ scale mentioned above, see Table 9, and if they had any discomfort, they were asked to choose another time for the experiment in order not to affect the results. When the subjects were in good physical and mental condition, they would sit still for 3 min in the driving simulator to eliminate the fatigue state and record the EEG signals in this state for baseline data.

**Table 9:** SSQ scales.

Symptomatic	None	Mildly	Moderately	Severity
1. Wearily	O	O	O	O
2. Headaches	O	O	O	O
⦙	⦙	⦙	⦙	⦙
15. Difficulty focusing vision	O	O	O	O
16. Whole-body discomfort	O	O	O	O

Evoked stage:

Subjects reported their MSS ratings after completing a driving task on a selected road using the simulator while the experimenter made EEG markings.

Modulation stage:

The subjects stopped the driving task, the experimenter played the corresponding type of music to the subjects in the modulation group (60 s) for modulation, and the subjects in the control group took the natural recovery measures to relieve the motion sickness. All subjects reported the level of motion sickness after 60 s, and the experimenter labeled the EEG data.

**Figure 4:**
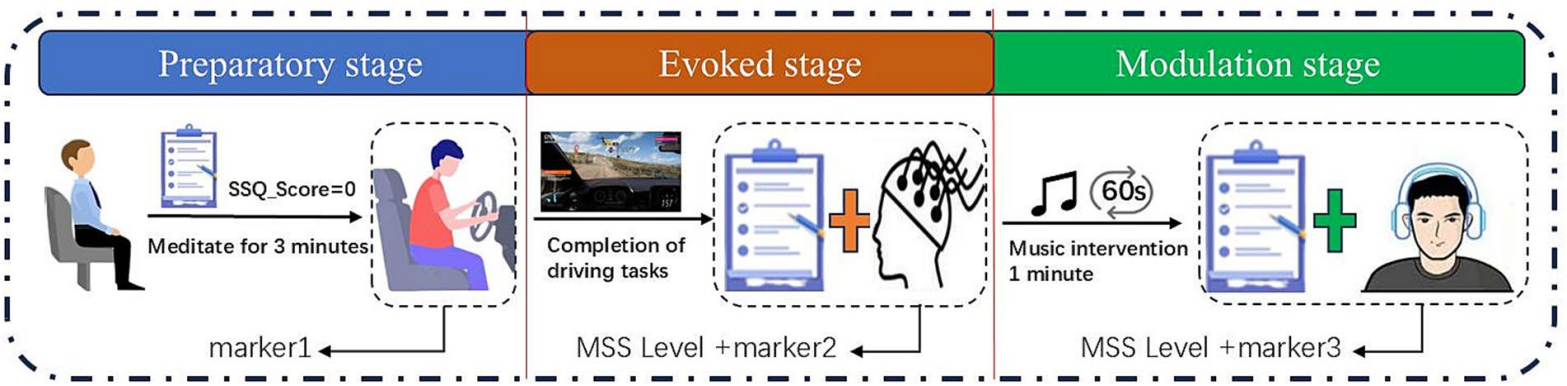
Experimental procedure for motion sickness induction and relief.

The experimental flow is shown in Figure 4.

#### 2.3.3 Ethical statement

The studies involving humans were approved by Ethics Committee of Chongqing University of Arts and Sciences (Approval No. CQWL202541). The studies were conducted in accordance with the local legislation and institutional requirements and adhere to the Declaration of Helsinki. The participants provided their written informed consent to participate in this study. Written informed consent was obtained from the individual(s) for the publication of any potentially identifiable images or data included in this article.

### 2.4 Data processing and analysis

**Figure 5:**
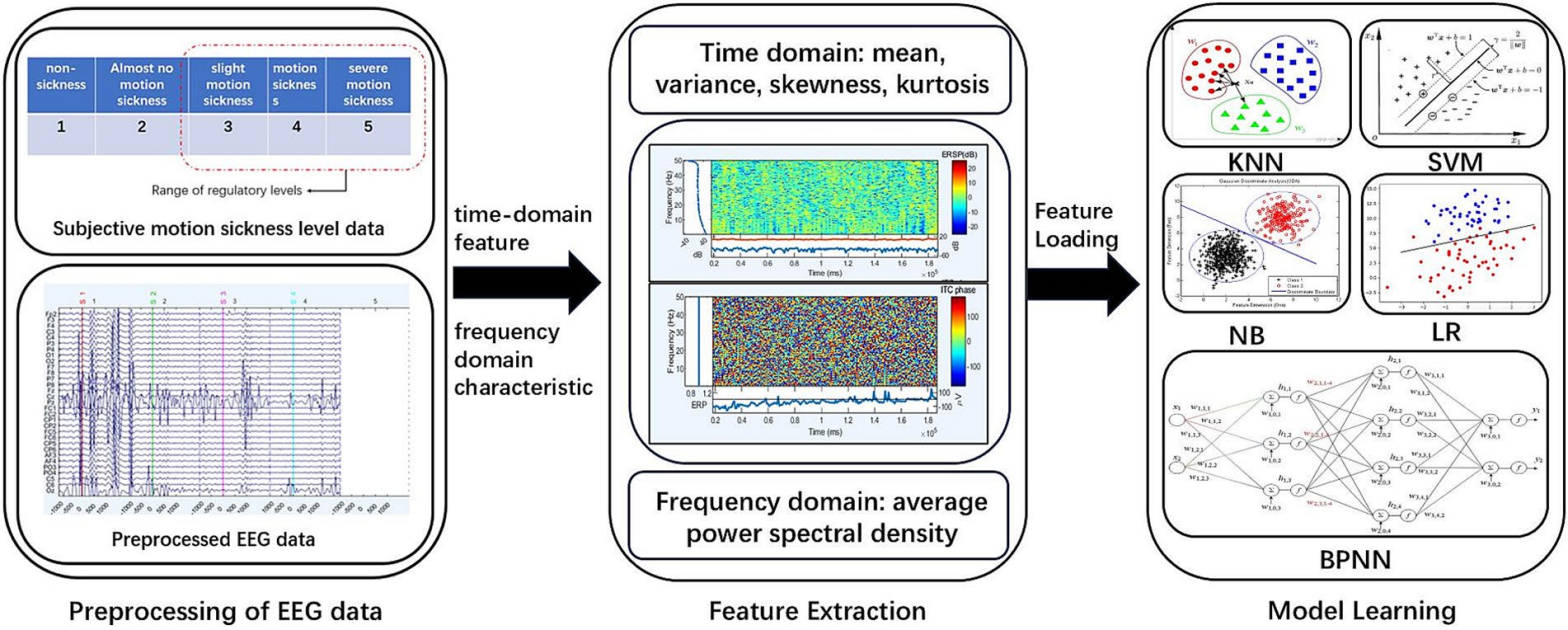
EEG data processing and analysis process.

The process of analyzing, processing and classifying EEG data related to motion sickness is shown in Figure 5, which contains: data preprocessing, feature extraction and model training (Amin et al., 2015). Feature extraction mainly focuses on time domain and frequency domain features, and finally the extracted features are organized and loaded into the model for training and classification to obtain classification results.

#### 2.4.1 Data preprocessing

**Figure 6:**
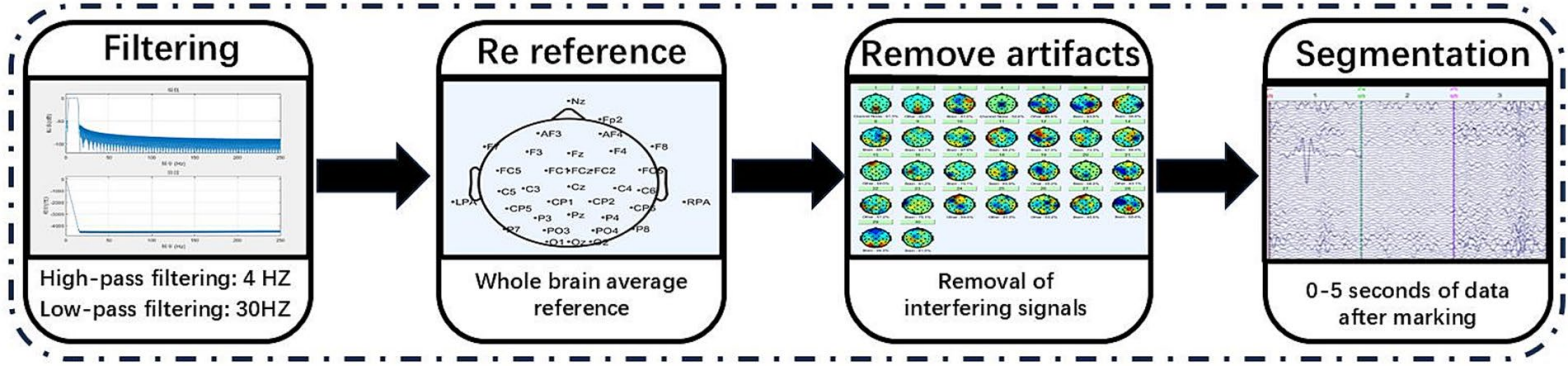
EEG data preprocessing process.

EEG signals are susceptible to a variety of noise interferences during acquisition, such as ophthalmoscopic, electromyographic, industrial frequency noise (50 Hz/60 Hz), and device drift (Usakli, 2010), which can seriously affect the accuracy of subsequent analysis. Therefore, data preprocessing is a key step in EEG analysis, aiming to improve the signal-to-noise ratio and retain effective physiological information. Commonly used preprocessing methods include band-pass filtering to remove high-frequency noise and low-frequency drift, independent component analysis to separate artifacts, bad-conduct interpolation, and segment alignment. Through preprocessing, the robustness of the feature extraction and classification models can be significantly improved, laying a reliable data foundation for subsequent research (Musthafa et al., 2024). The flowchart of the preprocessing of EEG signals in this paper is shown in Figure 6.

#### 2.4.2 Feature extraction

(1) Time-domain features: EEG time-domain features, as a kind of temporal signal, can reflect the amplitude and statistical properties of the signal over time, and are used to analyze the basic morphology and fluctuation patterns of EEG waveforms (Hjorth, 1970). The time domain features extracted in this study include: mean, variance, skewness, kurtosis.

(a) Mean: the average of the EEG signals, which is the sum of all the sampled values divided by the total number of points. The formula is as follows:

(2) $$\bar{X}=\frac{1}{N}\sum_{i=1}^{N}x_{i}$$

(b) Variance: EEG variance characterizes the degree of dispersion of the signal amplitude, reflecting the intensity of fluctuations in EEG amplitude, and can be used to assess the state of brain activity. The formula is as follows:

(3) $$\sigma^{2}=\frac{1}{N}\sum_{i=1}^{n}{\left(x_{i}-\mu \right)}^{2}$$

(c) Skewness: EEG skewness reflects the asymmetry of the signal amplitude distribution, with positive skewness indicating more high amplitudes and negative skewness vice versa, and can be used to detect abnormal EEG activity. The formula is as follows, where б is the standard deviation:

(4) $$S=\frac{1}{N-1}\sum_{n=1}^{N}{\left(\frac{x(n)-\mu }{\sigma }\right)}^{3}$$

(d) Kurtosis: EEG kurtosis reflects the sharpness of the signal amplitude distribution, with high values suggesting abnormal transient activity (e.g., epileptic waves) and low values indicating a smooth rhythm. The formula is as follows:

(5) $$K=\frac{\sum_{n=1}^{N}{\left(x(n)-\mu \right)}^{2}}{N}$$

(2) Frequency domain features: EEG frequency domain features reflect the distribution of signal energy in different frequency bands and are used to analyze brain rhythmic activities (Al-Fahoum and Al-Fraihat, 2014).

(a) Power Spectral Density(PSD): indicates the signal power per unit frequency band, and the decomposition of EEG signals into frequency-domain energy distributions by Fourier transform is the core frequency-domain feature for portraying EEG rhythms. First, the Fourier transform is utilized to convert the EEG signal from the time domain to the frequency domain.

In this study, the Welch mean periodogram method was used to calculate the PSD of EEG data in order to analyze the characteristics of neural oscillations in different frequency bands. Five seconds after each marker was divided into five 1-s-long non-overlapping time windows to improve the temporal resolution and reduce the effect of transient noise, and a Hanning window was used for windowing to reduce spectral leakage and improve the accuracy of spectral estimation. Regarding the core parameters of PSD calculation, the number of FFT points is set to 256 to ensure the balance between spectral resolution and computational efficiency. A 50% overlap rate is used to enhance spectral smoothness and reduce the estimation variance.

The PSD was calculated using the Pwelch function in MATLAB software with the mathematical expression:


(6) $${\hat{P}}_{\mathrm{xx}}(f)=\frac{1}{K}\sum_{k=1}^{K}|\frac{1}{L}\sum_{n=0}^{L-1}x_{k}(n)\cdot \omega (n)\cdot e^{-j2\mathrm{\pi fn}}|2$$

In Equation:

$x_{k}(n)$ is the kth segment signal.

ω(n) is the Hanning window function.

L is the window length.

K is the total number of segments.

In this study, the average power density of two bands-theta band (4–8 Hz) and alpha band (8–13 Hz)-was extracted to reflect the neural oscillation patterns in different cognitive states, in which theta-band PSD is mainly related to memory encoding and attentional modulation, whereas alpha-band PSD can reflect the resting-state brain activity and is related to inhibitory control (Li et al., 2024). Finally, in order to reduce the influence of single-channel noise, the arithmetic mean of the PSDs of all effective channels in each brain region was taken to obtain the representative spectral characteristics of the brain region.

By combining time-and frequency-domain features, it is possible to comprehensively characterize the neural response induced by motion sickness and improve the accuracy of state identification and degree assessment.

#### 2.4.3 Classification modeling based on EEG signals

In this experiment, the data from the preparation stage and the evoked stage were selected to form the dataset for building the classification model, and all the subjects had a halo level of 1 in the preparation stage, and the evoked stage halo level of the subjects in the baseline group was stopped after the evoked stage halo level reached 2. Then the baseline sample cases were 30 for level 1, 10 for level 2, 8 for level 3, 6 for level 4, and 6 for level 5. To enhance the sensitivity of the model to the time-varying features of the EEG signal and to alleviate the small-sample limitation, the present study used a sliding-window strategy to segment the 5-s data after event labeling in 1-s steps (1-s window length, 4-s overlap). This method expands the original data volume by 5 times while maintaining the event-related temporal structure, and optimizes the model’s ability to generalize to individual response latencies by translational alignment of local time windows (Zhou et al., 2025).

The EEG data acquisition channels used were Fp2, AF3, AF4, F7, F3, Fz, F4, F8, FC5, FC1, FC2, FC6, C5, C3, Cz, C4, C6, CP5, CP1, CP2, CP6, P7, P3, Pz, P4, P8, PO3, PO4, O1, Oz, and O2, totaling 31 EEG channels. All EEG channels were categorized into 5 brain regions (frontal pole region, frontal lobe region, central region, parietal lobe region, and occipital lobe region) according to their locations.

**Figure 7:**
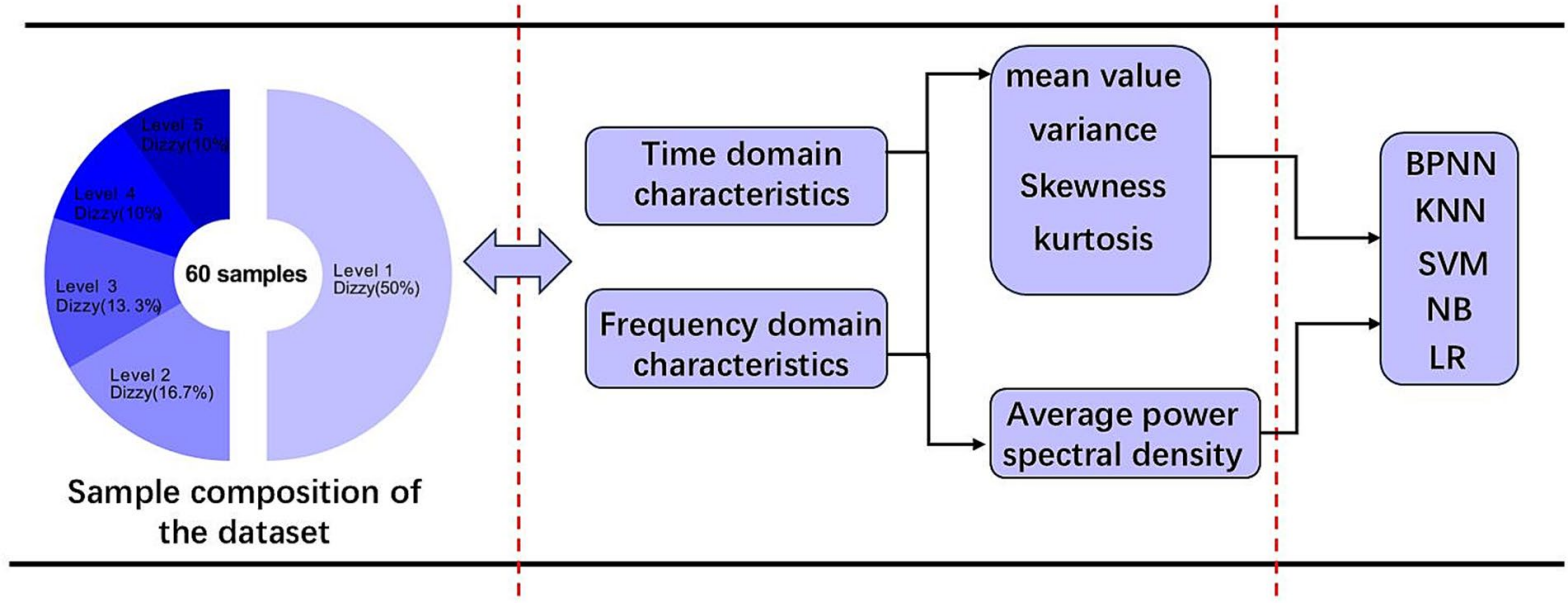
Overview of the five classification modeling.

Multidimensional features of five brain regions were extracted from the EEG signals, instantaneous domain statistical features (mean, variance, skewness, and kurtosis) as well as frequency domain features (power spectral densities in theta and alpha frequency bands). These features can comprehensively characterize the temporal and spatial dynamics of brain activity in the motion sickness state. Based on the extracted features, this study systematically compares the performance of traditional machine learning methods and deep learning models in motion sickness state recognition, providing an experimental basis for the establishment of an optimal classification prediction model. The models involved include five models, including BP neural network, K nearest neighbor (KNN), support vector machine (SVM), plain Bayes (NB) and logistic regression (LR). An overview of the model for classifying motion sickness levels based on EEG features is shown in Figure 7.

#### 2.4.4 Model evaluation

In this study, the performance of the motion sickness prediction model was evaluated and the model with the highest total score was set as the final model. Four metrics were used to evaluate the models, including accuracy, precision, recall, and F1 score. The calculation process of these metrics is as follows:


(7) $$\text{Accuracy}=\frac{\mathrm{TP}+\mathrm{TN}}{\mathrm{TP}+\mathrm{TN}+\mathrm{FP}+\mathrm{FN}}$$

(8) $$\text{Precision}=\frac{\mathrm{TP}}{\mathrm{TP}+\mathrm{FP}}$$

(9) $$\text{Recall}=\frac{\mathrm{TP}}{\mathrm{TP}+\mathrm{FN}}$$

(10) $$F1-\text{score}=\frac{2\cdot \text{Precision}\cdot \text{Recall}}{\text{Precision}+\text{Recall}}$$

where TP is the sample correctly predicted by the model to be in the positive category, TN is the sample correctly predicted by the model to be in the negative category, FP is the sample incorrectly predicted by the model to be in the positive category, and FN is the sample incorrectly predicted by the model to be in the negative category.

## 3 Result

### 3.1 Model evaluation results

**Figure 8:**
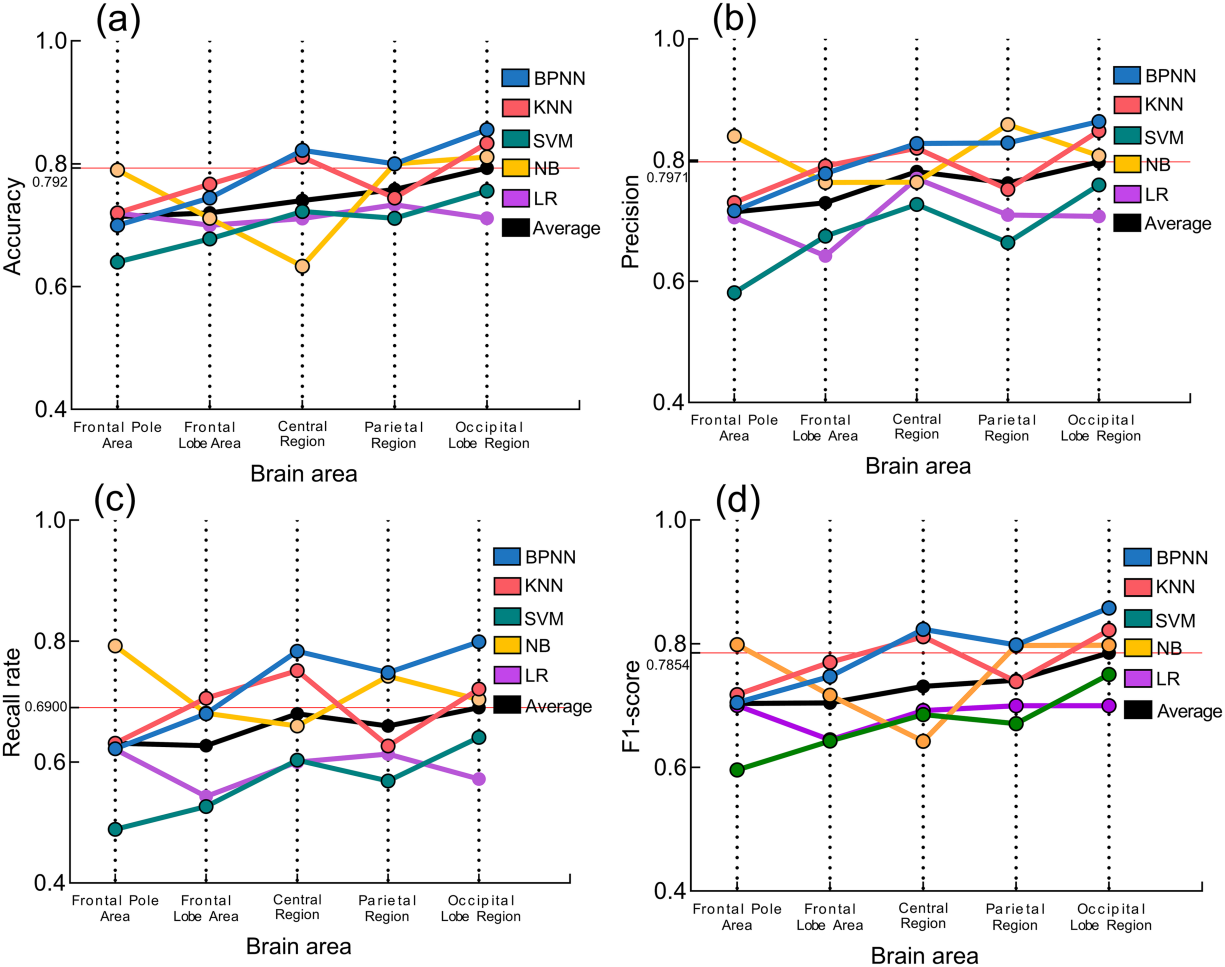
Performance of the five models. **(a)** Accuracy; **(b)** Precision; **(c)** Recall rate; **(d)** F1-score.

The performance of each model in terms of is shown in Figure 8. Observing the black folded line in the figure, the average scores of accuracy, precision, recall, and F1 score of the five models all achieved the maximum in the occipital lobe area, which to some extent indicates that the EEG signals in the occipital lobe area are closely related to motion sickness, and is the same as the conclusion drawn by Chen et al. (2010). Since the BPNN model under the occipital lobe area is again superior to the other four models, the BPNN model under the occipital lobe area was finally selected as the final model for occupant motion sickness recognition.

**Figure 9:**
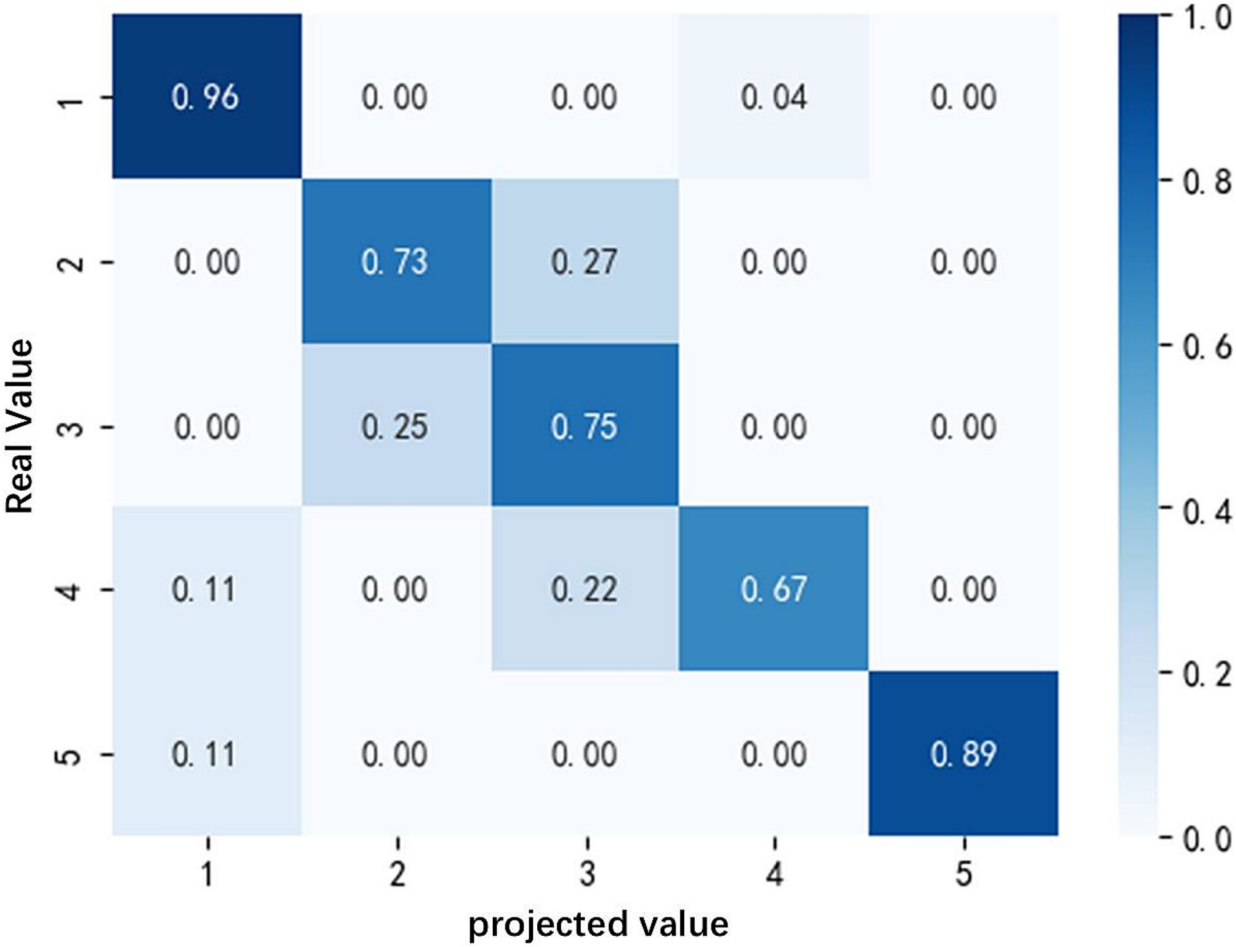
Classification confusion matrix based on BPNN models.

The confusion matrix of the BPNN-based motion sickness recognition model is shown in Figure 9.

The BPNN model in this study uses a single hidden layer structure (100 neurons with ReLU activation), the input layer receives 6 EEG features and the output layer corresponds to 5 motion sickness levels. After 1980 iterations of training, the model successfully converged and achieved the highest accuracy (85.6%) in the test set. Its stratified sampling validation and standardized preprocessing ensured generalization reliability. Preservation of the completed motion sickness emotion model classified by the BPNN algorithm using EEG features trained under the occipital lobe area prepares the model for further modulation analysis.

### 3.2 Music modulation results

In order to analyze the relief effect of the four music types on motion sickness and compare it with the state after natural recovery, the EEG features in the occipital lobe area after the modulation of each type of music (modulation group) as well as after the natural recovery (control group) were inputted into the constructed and completed recognition model of motion sickness, and we used the relief effect index *η* as an evaluation criterion, with the formula as follows:


(11) $$\eta =-\frac{\left(R_{A}-R_{B}\right)}{R_{B}}\times 100\%$$

Among them:

$R_{A}$ is the post-relief halo rating; $R_{B}$ is the pre-relief halo rating.

**Figure 10:**
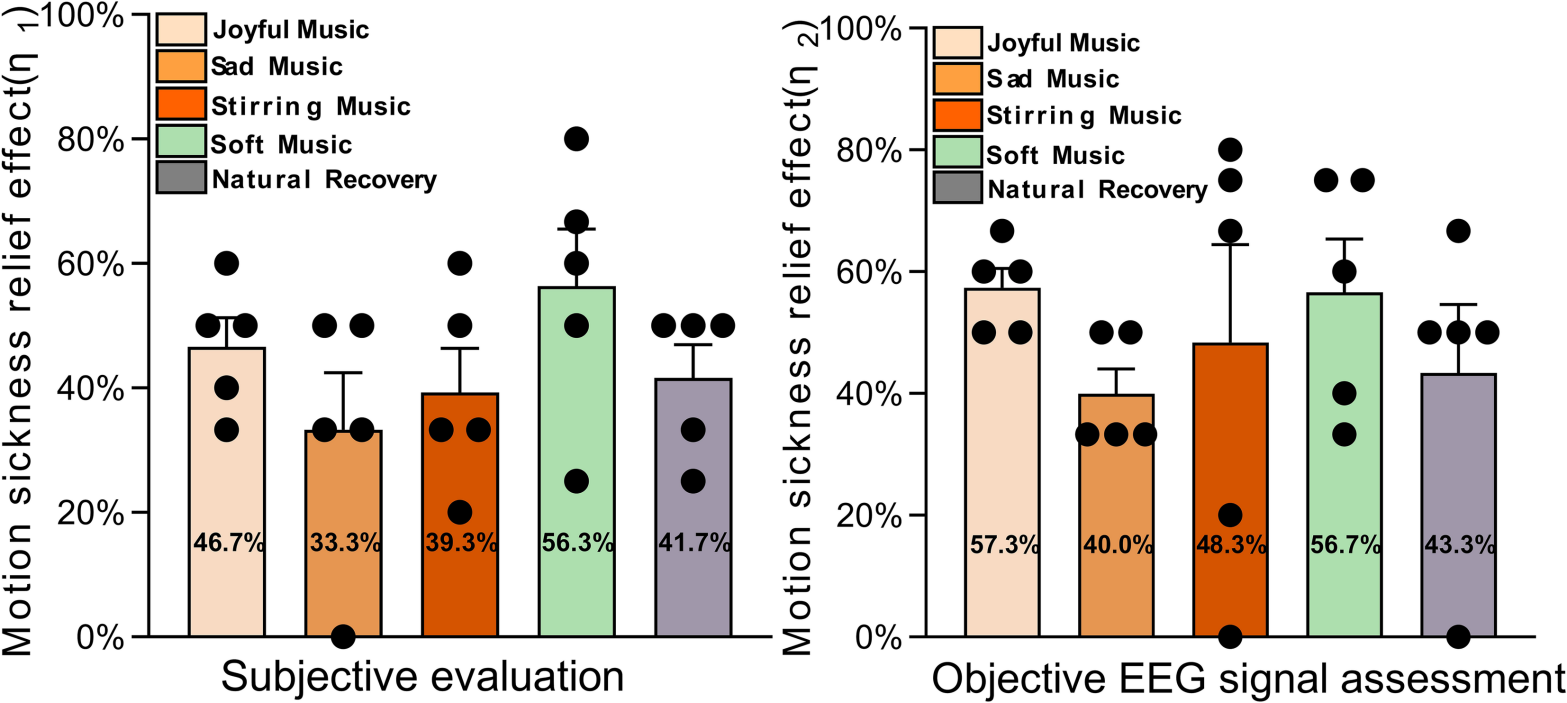
Mitigation effect scores based on subjective and objective data.

The mitigation score $\eta_{2}$ based on objective EEG data and the mitigation score $\eta_{1}$ based on subjective evaluations collected during the experiment were organized as shown in Figure 10.

As shown in Figure 10, there is a correlation between the subjective relief scores of 25 subjects in 5 groups and the objective relief scores based on EEG data. In the subjective scores, the motion sickness relief effect of soft music and joyful music was better than that of natural recovery, while sad music and stirring music were not as good as that of natural recovery; in the objective scores based on EEG data, the motion sickness relief effect of joyful music, soft music and stirring music were better than that of natural recovery, and similarly, sad music was not as good as that of relief under natural recovery. Comparing the subjective and objective motion sickness relief scores, it can be concluded that joyful music and soft music have better motion sickness relief effects, while sad music is less effective and stirring music is moderate. A correlation analysis was performed between subjective relief effects and alpha power spectral density in the occipital lobe region. The results showed a close correlation between the two (*p* < 0.05). When the subjects’ motion sickness was relieved, there was a significant increase in alpha wave power spectral density.

### 3.3 The analysis and verification of Kolmogorov-Chaitin complexity for EEG

Currently, the concept of Kolmogorov-Chaitin(KC) complexity we use actually refers to Lempel-Ziv complexity. KC complexity is a data processing method based on data coarse-graining (Odan, 2024), and the process of different coarse-graining is called N-valuing. In this paper, we focus on the KC complexity of binarization. After coarse-graining, the LZ complexity becomes insensitive to noise and is very suitable for processing bioelectric signals similar to EEG (Liu et al., 2010). It has been shown that the KC complexity of EEG signals is closely related to mental fatigue, and it has been experimentally found that the value of KC complexity gradually decreases as mental fatigue increases in the human brain (Zhang and Zheng, 2008).

We will continue to study the EEG KC complexity of the occipital lobe region corresponding to the three channels (O1, Oz, O2). The corresponding KC complexity of the occipital lobe region will be extracted as follows.

Binarization: the mean of the EEG signal within each time window is first calculated and the signal is converted to a binary sequence.


(12) $$S_{i}=\{\begin{array}{c} 1,\text{IF}\;x_{i}\geq \text{Mean}\\ 0,\text{IF}\;x_{i}<\text{Mean} \end{array}$$

where $x_{i}$ is the EEG signal sampling power and $S_{i}$ is the binarized sequence.

Computational complexity c(n) : subsequently traverse the binary sequence and count the num (Zhang et al., 2016) ber of non-repeating sub-patterns in it c(n) , Initialize c(n) =1, initial sub-patterns are $S_{1}$ , expand the substring step by step, if the new substring cannot be duplicated from the existing pattern, c(n) increases, and after traversing the whole sequence, the final c(n) is obtained.

Normalized KC complexity: logarithmic normalization is used to avoid sequence length effects.


(13) $$\mathrm{KC}=\frac{c(n)}{b(n)}$$

(14) $$b(n)=\frac{n}{\log_{2}n}$$

n is the sequence length, and the normalized EEG KC complexity takes values in the range (0,1).

**Figure 11:**
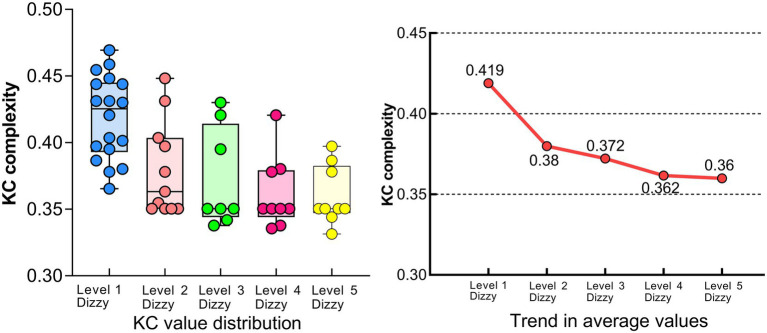
EEG KC complexity for each level of motion sickness.

Finally, the average EEG KC complexity under different levels of motion sickness was calculated. Figure 11 shows the distribution of EEG KC complexity of subjects under different levels and the corresponding mean values.

It can be seen that when the subjects were in a calm state, the overall distribution of KC values in the occipital lobe area was high, and after the occurrence of motion sickness, the KC complexity in the occipital lobe area of the subjects generally appeared to have numerous low level values; in the level 2–5 motion sickness state, there were a small number of high level KC values, which may be due to the individualization of the differences in the subjects; the KC complexity in the occipital lobe area of the subjects was close to the same level as a whole, and the trend of the change in the mean value can be seen that it still showed a decreasing Trend (Liu et al., 2020).

**Figure 12:**
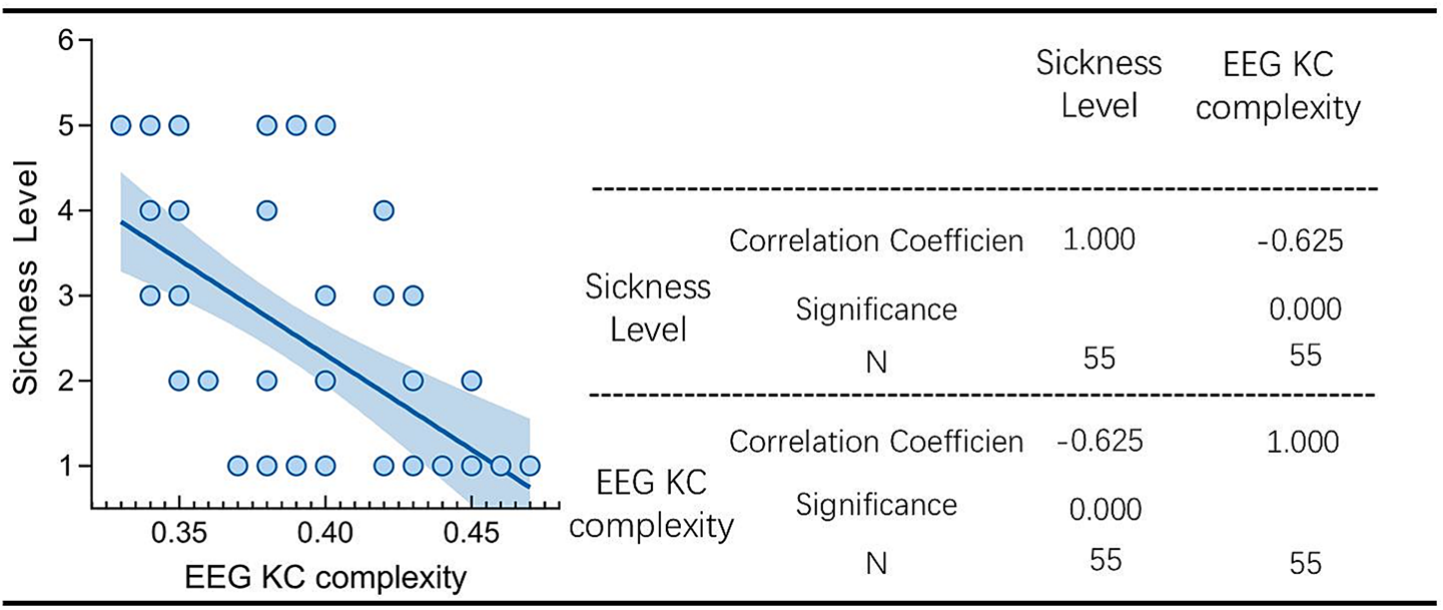
Correlation analysis of motion sickness level and EEG KC complexity.

Subsequently, the correlation analysis test between the degree of dizziness and the KC complexity of the EEG signals in the occipital region was performed using the prism software, and the results are shown in Figure 12.

As can be seen from the figure, the correlation coefficient between motion sickness level and EEG KC complexity in occipital lobe area was −0.625, and the two of them showed a significant negative correlation, i.e., it indicates that, when the degree of motion sickness is higher, the EEG KC complexity in occipital lobe area decreases at this time. The occipital lobe area in which O1, O2, and Oz are located has a very high sensitivity to motion sickness, which is not only in line with the conclusions demonstrated in the previous sub-brain area and sub-model analyses, but also further verifies the accuracy of the model based on the recognition of motion sickness. Occipital lobe regions to recognize the accuracy of the motion sickness recognition model, and at the same time re-validates the conclusions drawn by Chen et al.

## 4 Discussion

This study used an objective assessment system based on electroencephalographic signals to reveal the differential effects of different music genres on the alleviation of motion sickness. The results showed that joyful music and soft music had better alleviating effects on motion sickness, with alleviation rates of 57.3 and 56.7%, respectively; while sad music had a lower alleviation effect (40%) than natural recovery (43.3%). The BPNN recognition model constructed based on these findings (with an accuracy rate of 85.6% in the occipital lobe region) further validated the strong association between music intervention effects and neural activity in the occipital lobe region, providing quantifiable metrics for real-time music regulation.

### 4.1 Discussion of model prediction results

This study compared the performance of five machine learning models in classifying electroencephalographic signals and found that the BP neural network model in the occipital lobe region performed best in terms of accuracy, precision, recall, and F1 score.

Presenting such results may stem from the synergistic effect of physiological mechanisms and algorithmic properties. As a core area for visual information processing, the occipital cortex plays a key role in the development of motion sickness. Previous studies have shown that visual-vestibular signal conflict is a major factor inducing motion sickness, while the occipital area receives and integrates visuomotor information, and its EEG activity can directly reflect an individual’s physiological response to visual stimuli. When subjects were exposed to different types of relieving music, the power changes of alpha (8–13 Hz) and theta (4–8 Hz) waves in the occipital region may reflect the effect of music modulation on visual information processing, which provides highly discriminative feature inputs for the BPNN model.

The performance advantage of BP neural network is attributed to its deep nonlinear modeling ability. BPNN can effectively capture the complex dynamic features of EEG signals in occipital region under music intervention, and its adaptive weight adjustment mechanism shows stronger robustness to individual differences and noise interference, which is significantly better than the shallow model. In contrast, KNN and SVM may be limited by the local similarity metric and linear kernel constraints, which make it difficult to adequately characterize the high-dimensional nonlinear distributions of features related to motion sickness.

### 4.2 Intervention effects of music on motion sickness

This study found that soft and joyful music was significantly more effective than sad music in relieving motion sickness, while stirring music had a moderate effect. Both subjective and objective data consistently supported this conclusion. This finding not only validated existing research but also revealed, to a certain extent, the specific mechanisms involved in motion sickness regulation.

Subjective scores showed that the motion sickness relief effects of soft and joyful music were significantly better than natural recovery, a result that was further verified in the EEG data. The soothing rhythm of soft music may reduce the symptoms of nausea and dizziness associated with motion sickness by modulating the autonomic nervous system and reducing sympathetic excitability. In addition, soft music may enhance alpha wave (8–13 Hz) activity in the occipital region, promote the formation of a relaxed state in the brain, and alleviate visual-vestibular conflicts. In contrast, the positive effects of joyful music may stem from its rhythmic motivational effect, which elevates emotional states by activating brain reward systems (e.g., the nucleus ambiguus), thereby distracting individuals from dizziness discomfort. Subjective and objective data consistently show that sad music is even less effective in relieving motion sickness than natural recovery. This phenomenon may stem from an emotional resonance effect—the subdued melodies of sad music may intensify negative emotional experiences, superimposing them on the discomfort of motion sickness and thus exacerbating subjective discomfort. Neurologically, sad music may inhibit emotion regulation in the prefrontal-limbic system, making it more difficult for individuals to recover from motion sickness, as has been demonstrated in previous laboratory studies. The study was conducted in the laboratory of the University of California, Berkeley, and the United States (Li et al., 2025).

### 4.3 Relationship between EEG KC complexity and motion sickness levels

In this study, we analyzed the Kolmogorov-Chaitin (KC) complexity changes of EEG signals in the occipital lobe area under different motion sickness states and found that the degree of motion sickness showed a significant negative correlation with KC complexity (r = −0.625, *p* < 0.05). These results reveal the neural mechanism of motion sickness from the perspective of nonlinear dynamics and provide theoretical support for motion sickness recognition models based on the occipital lobe area.

In the calm state, the visual cortex’s EEG activity exhibited higher KC complexity, reflecting its high information complexity and nonlinear dynamic properties during normal visual information integration. With the onset of dizziness symptoms, KC values decreased significantly, indicating weakened dynamic complexity in the visual cortex.

### 4.4 Limitations and future work

First, although experiments were conducted in a simulated environment, the current sample size is still relatively small. Analysis indicates that the statistical power (29%) under the current sample grouping has not yet reached conventional standards, which to some extent reduces the certainty of the conclusions. Second, during the data processing stage, only commonly used features were extracted for analysis, and the contribution of different features to motion sickness classification was not evaluated, which may introduce redundant features and affect the model’s interpretability and generalization ability. Finally, the experiments were conducted using a driving simulator, which inevitably differs from real-world driving environments, potentially affecting the intensity of motion sickness induction and EEG response patterns. The age range of participants was narrow (20–30 years old), while susceptibility to motion sickness may vary with age.

In subsequent studies, to further validate the conclusions, we will expand the experimental sample size to achieve statistical power and construct a more comprehensive set of EEG features in the experiment. We will use feature selection algorithms to identify the most discriminative feature subsets to improve model efficiency. We will conduct real-vehicle experiments to assess the external validity of the simulator experiment conclusions.

## 5 Conclusion

In summary, this study constructed and screened various motion sickness recognition models by integrating EEG signal analysis and machine learning techniques, and systematically explored the intervention effects of different music types on motion sickness. The results showed that the five types of features extracted by sub-brain regions (mean, variance, skewness, kurtosis, and power spectral density) were inputted into a variety of models and performed better in the occipital lobe area, and the BPNN model was again the best. BPNN model was again optimal; subjective and objective data consistently showed that: gentle music and cheerful music both significantly alleviated motion sickness symptoms; while sad music may exacerbate discomfort through emotional resonance. Notably, agitated music showed context-dependent effects, which were effective in objective indicators but poor in subjective experience. Based on the above conclusions, in the future, we can monitor the changes of motion sickness of the occupants in real time and play suitable music types according to the state of motion sickness, so as to utilize the alleviating effect of music to help the occupants maintain a good physical state, thus enhancing the comfort of the occupants.

## Data Availability

The raw data supporting the conclusions of this article will be made available by the authors, without undue reservation.
